# Influences of Shifted Vegetation Phenology on Runoff Across a Hydroclimatic Gradient

**DOI:** 10.3389/fpls.2021.802664

**Published:** 2022-01-04

**Authors:** Shouzhi Chen, Yongshuo H. Fu, Xiaojun Geng, Zengchao Hao, Jing Tang, Xuan Zhang, Zongxue Xu, Fanghua Hao

**Affiliations:** ^1^College of Water Sciences, Beijing Normal University, Beijing, China; ^2^Plants and Ecosystems, Department of Biology, University of Antwerp, Antwerp, Belgium; ^3^Department of Physical Geography and Ecosystem Science, Lund University, Lund, Sweden; ^4^Terrestrial Ecology Section, Department of Biology, University of Copenhagen, Copenhagen, Denmark; ^5^Center for Permafrost (CENPERM), University of Copenhagen, Copenhagen, Denmark

**Keywords:** vegetation phenology, runoff, climate change, semi-humid and humid regions, river basins

## Abstract

Climate warming has changed vegetation phenology, and the phenology-associated impacts on terrestrial water fluxes remain largely unquantified. The impacts are linked to plant adjustments and responses to climate change and can be different in different hydroclimatic regions. Based on remote sensing data and observed river runoff of hydrological station from six river basins across a hydroclimatic gradient from northeast to southwest in China, the relative contributions of the vegetation (including spring and autumn phenology, growing season length (GSL), and gross primary productivity) and climatic factors affecting the river runoffs over 1982–2015 were investigated by applying gray relational analysis (GRA). We found that the average GSLs in humid regions (190–241 days) were longer than that in semi-humid regions (186–192 days), and the average GSLs were consistently extended by 4.8–13.9 days in 1982–2015 period in six river basins. The extensions were mainly linked to the delayed autumn phenology in the humid regions and to advanced spring phenology in the semi-humid regions. Across all river basins, the GRA results showed that precipitation (*r* = 0.74) and soil moisture (*r* = 0.73) determine the river runoffs, and the vegetation factors (VFs) especially the vegetation phenology also affected the river runoffs (spring phenology: *r* = 0.66; GSL: *r* = 0.61; autumn phenology: *r* = 0.59), even larger than the contribution from temperature (*r* = 0.57), but its relative importance is climatic region-dependent. Interestingly, the spring phenology is the main VF in the humid region for runoffs reduction, while both spring and autumn growth phenology are the main VFs in the semi-humid region, because large autumn phenology delay and less water supply capacity in spring amplify the effect of advanced spring phenology. This article reveals diverse linkages between climatic and VFs, and runoff in different hydroclimatic regions, and provides insights that vegetation phenology influences the ecohydrology process largely depending on the local hydroclimatic conditions, which improve our understanding of terrestrial hydrological responses to climate change.

## Introduction

Climate warming has substantially extended the length of vegetation growing period in temperate and boreal zones (Piao et al., 2019; Menzel et al., 2020) and sequentially resulted in a widespread increase of leaf area index (LAI) over the past decades (Zhu et al., 2016; Piao et al., 2020). Studies have shown that these phenological changes have affected the regional climate through altered biogeochemical cycles and water fluxes and changed the physical properties of the land surface (Peñuelas and Filella, 2009; Wang et al., 2019; Xu et al., 2020). However, it remains unclear whether there is a linkage between the changes of plant phenology to the runoff dynamics (Lei et al., 2014; Geng et al., 2020), especially its effect may be largely different in different hydroclimatic regions. Therefore, it is essential to investigate the effect of vegetation phenology and growth on runoffs across river basins with different climates to improve our understanding of terrestrial water cycles under climate change.

Vegetation plays an important role in the hydrological cycle through intercepting precipitation, transpiring water to the atmosphere (Kim et al., 2014), and increasing the water holding capacity of the soil and surface roughness (Myneni et al., 1997; Zhang et al., 1999; Rodriguez-Iturbe, 2000; Yang et al., 2009; Piao et al., 2020). For example, warming-caused increase in LAI has attributed to the increased transpiration at regional and global scales in 1982–2011 (Li Y. et al., 2018), although the stomatal conductance of leaves was significantly reduced due to increased CO_2_ concentration (Ainsworth and Rogers, 2007; Yang et al., 2019). The recent studies have found that under the interactive effect of these two aspects, i.e., increase in LAI and decrease in stomatal conductance, the river runoff at basin scales has experienced generally decreasing trends (Xu et al., 2018; Gao et al., 2019; Li et al., 2019), although the increased trends were also reported in some regions (Xi et al., 2018). This inconsistent result may be related to the different roles of vegetation on water cycles at different climate zones. It has been well reported that the main factors influencing the hydrological cycle are meteorological factors (Menzel and Bürger, 2002; Kling et al., 2012), but the impacts of vegetation should not be ignored (Lei et al., 2014; Zhang et al., 2019), especially at the humid climates (Shen et al., 2013). However, to the best of our knowledge, there is still a lack of studies on the quantification of vegetation effects on river runoff across basins with hydroclimatic gradients.

Phenology determines the start and end time of vegetation growth and is highly sensitive to climate change (Myneni et al., 1997; Beck et al., 2006; Liang and Schwartz, 2009; Fu et al., 2015, 2019). Climate warming has advanced the spring phenology and delayed autumn phenology across the globe (Piao et al., 2019). On the one hand, earlier spring phenology promotes a longer growing season and can increase the period for plant transpiration (Geng et al., 2020; Wu et al., 2021), but earlier spring phenology may also induce water stress in summer, and thus limit transpiration loss and might increase the river runoffs (Liu Q. et al., 2018). On the other hand, delayed autumn phenology extends the growing season, potentially resulting in larger transpiration and might reduce the river runoff (Piao et al., 2020). However, how the spring and autumn phenology affects river runoff at different climatic zones and its relative importance is still largely unclear needs to be investigated.

Based on the long-term phenology and vegetation growth data, and runoff observations across six river basins ranging from subtropical to cold-temperate regions in China, testing our hypothesis that the climatic factors (CFs) determine the river runoffs and the vegetation factors (VFs), especially the vegetation phenology, is also affected the river runoffs, but its relative importance is climatic region-dependent. The relative contributions of the VFs [including spring and autumn phenology, growing season length (GSL), and gross primary productivity (GPP)] and CFs affecting river runoff across a hydroclimatic gradient were quantified by applying the gray relational analysis (GRA). The aim of this study was to (1) analyze the temporal–spatial changes in both phenology and river runoffs across hydroclimatic gradient river basins; (2) quantify potential influences of climatic and VFs on river runoff; and (3) explore the differences between humid and semi-humid regions in primary factors impacting river runoff.

## Materials and Methods

### Study Area Description

**FIGURE 1:**
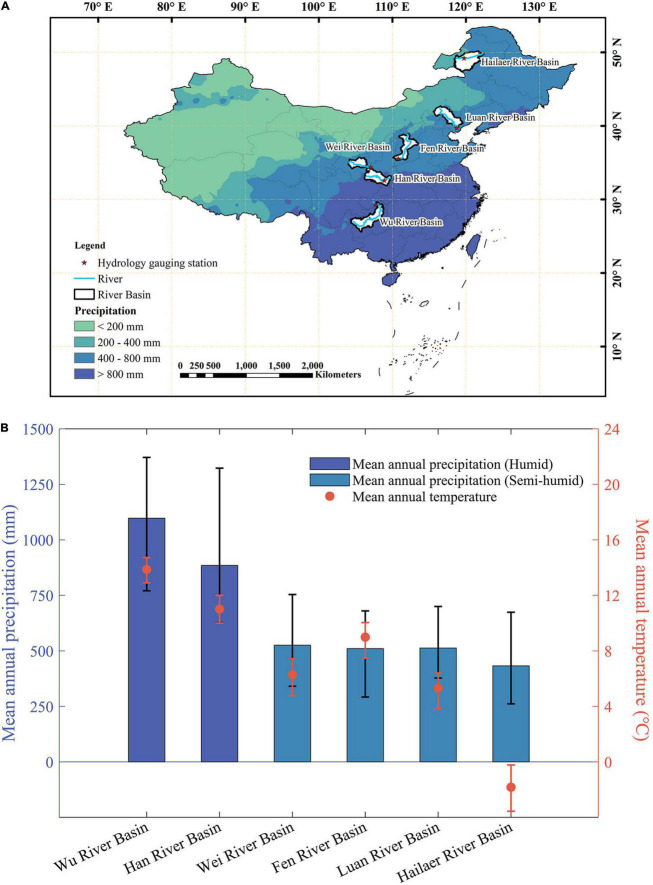
The location and basic climatic conditions of the six river basins during 1982–2015. **(A)** The spatial distribution of the six river basins, and the red pentacles indicate the hydrological stations. **(B)** The mean annual temperature (MAT) and mean annual precipitation (MAP) for each river basin. The error bars represent the one standard deviation.

Six river basins were selected across a hydroclimatic gradient, i.e., Wu and Hailaer river basins, changing from subtropical in the south to the cool temperature zones in the north of China (Figure 1A and Table 1). According to the mean annual precipitation (MAP), we divided the river basins into two groups (Figures 1A,B): humid river basins with MAP >800 mm, including Wu river basin (MAP = 1097.7 mm) and Han river basin (MAP = 885.1 mm) and semi-humid river basins (400 < MAP < 800 mm) including Wei river basin (MAP = 525.4 mm), Fen river basin (MAP = 510.0 mm), Luan river basin (MAP = 513.0 mm), and Hailaer river basin (MAP = 423.6 mm). The details of each selected river basin are shown in Table 1.

**TABLE 1:** The specific information of the six river basins.

River basin	Lon (°E)	Lat (°N)	Area (km^2^)	Climatic region	Gauging station	MAT (°C)	MAP (mm)
Wu	104.4–108.9	25.5–29.6	69479.4	Humid	Wulong	13.9	1097.7
Han	106.2–109.9	31.8–34.3	46773.9	Humid	Ankang	11.0	885.1
Wei	104.0–106.6	34.2–35.7	26118.9	Semi-humid	Linjiacun	6.3	525.4
Fen	110.1–113.5	35.3–39.0	45945.5	Semi-humid	Hejin	9.0	510.0
Luan	115.6–119.6	39.7–42.7	43529.0	Semi-humid	Luanxian	5.3	513.0
Hailaer	118.4–122.5	47.4–50.2	54535.6	Semi-humid	Hailaer	−1.8	432.6

*MAT, mean annual temperature; MAP, mean annual precipitation.*

### Datasets Description

The meteorological data, including daily precipitation, temperature, relative humidity, wind speed, and radiation with 0.1° × 0.1° spatial resolution from 1982 to 2015, were provided by the China Meteorological Forcing Dataset and were produced by the Institute of Tibetan Plateau Research, Chinese Academy of Sciences^1^ (He et al., 2020). The mean annual temperature (MAT) and precipitation were averaged across all pixels within each river basin during 1982–2015. The normalized difference vegetation index (NDVI) data were received from The Global Inventory Modeling and Mapping Studies NDVI3g dataset. This dataset is from Advanced Very High Resolution Radiometer (AVHRR), with bi-monthly temporal resolution and 8-km spatial resolution^2^ (Tucker et al., 2005). Gross primary productivity data with the 0.1° × 0.1° spatial resolution and 15-day interval were provided by the Global Land Surface Satellite (GLASS) program. This GLASS-GPP product was estimated using an improved EC-LUE model, which included four input variables: NDVI, photosynthetically active radiation, air temperature, and the Bowen ratio of sensible-to-latent heat flux.^3^ The phenology data including start of growing season (SOS) and end of growing season (EOS) with the 0.05° × 0.05° spatial resolution were provided by the Vegetation Index and Phenology Lab, The University of Arizona, in 2011^4^ (Zhang et al., 2003). Start of growing season and EOS were calculated by phenology extraction algorithm based on satellite vegetation index (AVHRR, SPOT, and MODIS). In this algorithm, two processes were applied to extract the phenology data: (1) application of smoothing and interpolation to obtain daily NDVI data and (2) use of the phenological phase extraction method based either on the threshold value or on the maximum rate of change (positive for SOS and negative for EOS) and the GSL which was defined as the number of days between the SOS and the EOS. The daily runoff data recorded by flowmeter from these six hydrological stations (located at the catchment outlet) from 1982 to 2015 were collected from the Hydrological Bureau of the Ministry of Water Resources of China. The monthly root-zone soil moisture (SMroot) data with 0.25° × 0.25° spatial resolution were obtained from the GLEAM_v3.3 Datasets,^5^ which were estimated by The Global Land Evaporation Amsterdam Model (GLEAM) (Martens et al., 2017). The monthly evapotranspiration (ET) data (with a spatial resolution of 0.1° × 0.1° between 1982 and 2015 covering the whole China) were produced by integrating remote sensing and eddy covariance data in a machine learning approach (model tree ensemble), and more details about this dataset can be found (Li X. et al., 2018). The land-use type data, which were based on Landsat 8 remote sensing images and generated by manual visual interpretation with 1-km spatial resolution in 1980, 1990, 1995, 2000, 2005, and 2015, are obtained from the Center for Resources and Environmental Science and Data.^6^ All data of each river basin were clipped and spatially interpolated using Exelis Visual Information Solution 5.3 and unified to the same temporal resolution with phenology data (annual) using MATLAB 2020a (MathWorks).

### Description of Statistical Methods

#### Temporal Trend Analysis Using Linear Regression and Analysis of Covariance

Linear regression analysis was used to analyze the temporal changes of phenological dates, i.e., the spring growth onset (SOS), the end of vegetation growth (EOS), and the GSL over the study period 1982–2015. The vegetation productivity during growing season (GPP_GS) was defined at the mean values of GPP between SOS and EOS. The temporal changes in climatic variables, i.e., temperature, precipitation, and runoff, were also estimated using linear regression. Due to the large differences in the basin size and also in the discharge magnitudes of the six basins, normalization, which we defined the runoff in the first year (i.e., 1982) as the standard and calculated the proportion of change of other years in each river basin, was applied to the data before analyzing and comparing the temporal trends in runoff among the six river basins. And we applied min–max normalization to factors with different units when comparing the variation of runoff, GSL_GS, and GSL.

To explore the difference in temporal dynamics of the runoff between humid and semi-humid regions, we applied the analysis of covariance (ANCOVA) to the runoff depth (ratio of runoff to watershed area) of humid and semi-humid river basins, following the previous study (Fu et al., 2014). All statistical analysis was conducted using the MATLAB 2020a (MathWorks) and IBM SPSS Statistics 20 (International Business Machines Corporation).

#### Gray Relational Analysis

To compare the relative importance of phenology, vegetation growth, and climate variables on the runoff, we applied the GRA, which was widely used in complex inter-relationships among the multiple performance characteristics by quantifying the gray relational grades (Wang et al., 1996; Zhu et al., 1996; Tan et al., 1998). The GRA can be described as follows (Cheng and Wang, 2004).

The reference sequence, i.e., runoffs in this study, is expressed as:


(1) $$x_{0}=\left(x_{0}\left(1\right),x_{0}\left(2\right),...,x_{0}\left(n\right)\right).$$

where *x*_0_ stands for reference sequence, and *n* indicates the number of records.

We denote the *m* sequences to be compared as:


(2) $$x_{i}=\left(x_{i}\left(1\right),x_{i}\left(2\right),...,x_{i}\left(n\right)\right),i=1,2,...,m.$$

where *x*_*i*_ represents the *i*th sequence to be compared and *m* is the total number of sequences to be compared.

We normalized the sequences to ensure that all of them are in the same order, and the normalized sequences can be denoted by:


(3) $$x_{i}^{{}^{*}}=\left(x_{i}^{{}^{*}}\left(1\right),x_{i}^{{}^{*}}\left(2\right),...,x_{i}^{{}^{*}}\left(n\right)\right),i=1,2,...,m.$$

where $x_{i}^{{}^{*}}$ represents the *i*th normalized sequence to be compared, and *m* is the total number of sequences to be compared.

The gray relational coefficient between the compared sequence, *x*_*i*_, and the reference sequence, *x*_0_, for the *j*th record, (*j* = 1, 2, … , *n*), is defined as:


(4) $$\xi_{i0}\left(j\right)=\frac{\Delta_{\min}+\rho \Delta_{\max}}{\Delta_{0i}\left(j\right)+\rho \Delta_{\max}},i=1,2,...,m,j=1,2,...,n,$$

where $\Delta_{0i}\left(j\right)=\left|x_{0}\left(j\right)-x_{i}^{{}^{*}}\left(j\right)\right|$ denotes the absolute difference between the reference sequence and the compared sequence; $\Delta_{\min}=\min_{i}\left\{\min_{j}\left|x_{0}\left(j\right)-x_{i}^{{}^{*}}\left(j\right)\right|\right\}$ and $\Delta_{\min}=\max_{i}\left\{\max_{j}\left|x_{0}\left(j\right)-x_{i}^{{}^{*}}\left(j\right)\right|\right\}$ are the minimum and maximum distances for all factors in all sequences. ρis the distinguishing coefficient which is defined in the range 0 ≤ ρ ≤ 1 and typically ρ = 0.5.

Then, the gray relational grade is derived as:


(5) $$\varepsilon_{i}=\frac{1}{n}\sum_{j=1}^{n}\xi_{0i}\left(j\right).$$

where ε_*i*_ is the gray relational grade for the *i*th factor and *n* is the number of records (years).

A larger gray correlation coefficient means a stronger correlation between the reference sequence and explanatory factor. We applied the GRA at each river basin and estimated the mean GRA coefficients across six basins for each impact factor. The reference sequence is river runoff, and the impact factors include CFs, i.e., precipitation, air temperature, soil moisture (SMroot), ET and radiation, and VFs, i.e., SOS, EOS, GSL, and GPP_GS.

## Results

### Temporal Changes of Climatic Variables and River Runoff During 1982–2015

**FIGURE 2:**
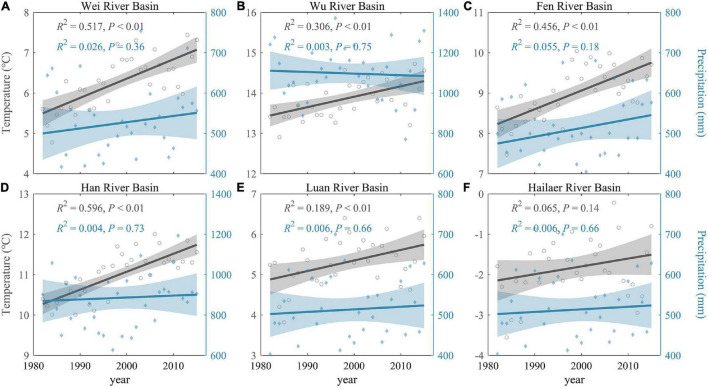
Temporal changes of annual climatic factors during 1982–2015 in all river basins. Temporal changes in MAT (gray) and MAP (blue) of six river basins **(A–F)** during 1982–2015. The gray-shaded areas represent 95% confidence interval.

During 1982–2015, the MAT of all the river basins increased significantly except the Hailaer river basin. The increment in Wei river basin (0.048°C/year, semi-humid region), Fen river basin (0.046°C/year, semi-humid region), and Han river basin (0.044°C/year, humid region) is larger than the rest of the basins (Figure 2 and Supplementary Figure 1). The MAP increased slightly without statistical significance for all six river basins. In addition, changes of MAP in the semi-humid regions (mean 1.69 mm/year) were larger than those humid regions (mean 0.87 mm/year).

**FIGURE 3:**
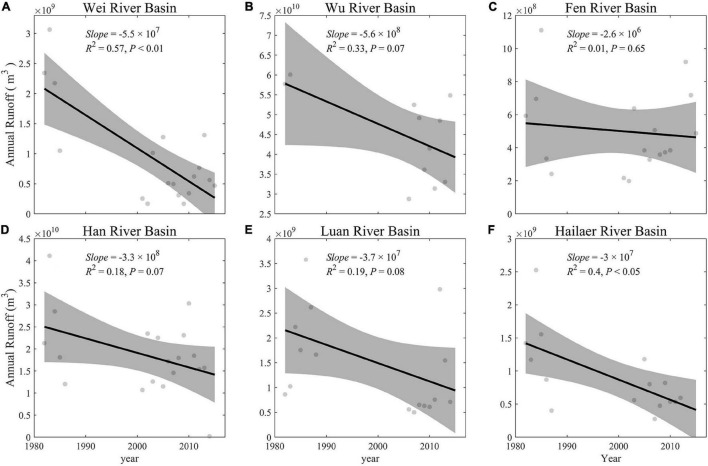
Temporal changes of the observed annual runoff in each river basin during 1982–2015 **(A–F)**. The gray-shaded area represents 95% confidence interval.

Over the study period, the river runoff of six river basins decreased with marginal significance (0.05 < *P* < 0.10, which is a confidence level close to significance) from 1982 to 2015, except for the Fen river basin (*P* = 0.65) (Pritschet et al., 2016) (Figure 3 and Supplementary Figure 2). Across the humid and semi-humid regions, the river runoff reduced significantly (*P* < 0.05), and the relatively reduced magnitudes were significantly larger in semi-humid river basins (normalized runoffs: 0.04 per year) than in humid basins (0.01 per year) (ANCOVA, *P* < 0.05, Supplementary Figure 3, and Supplementary Table 1).

### Spatial Pattern and Temporal Trends of Evapotranspiration

**FIGURE 4:**
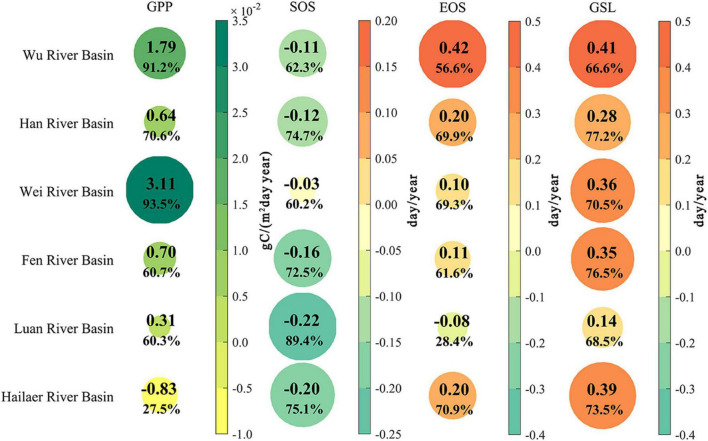
Temporal changes of annual vegetation growth (GPP) and phenology during 1982–2015 for the six river basins. The color and size represent the average rate and percentage of the area with the same changing direction. GPP, gross primary productivity; SOS, start of growing season; EOS, end of growing season; GSL, growing season length.

**FIGURE 5:**
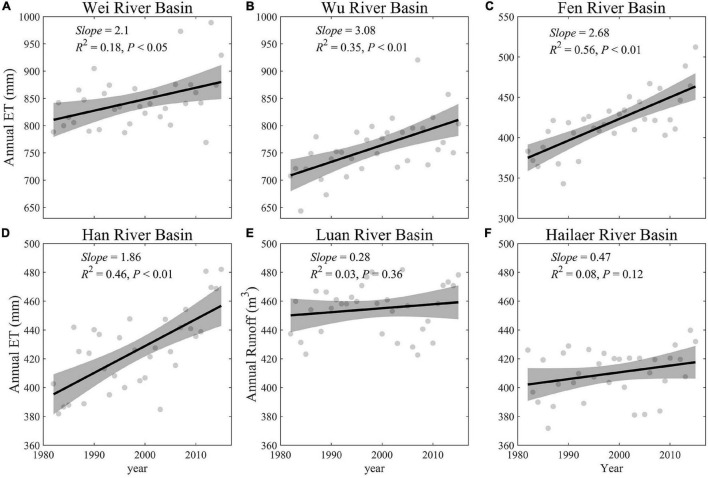
Temporal changes of the observed annual evapotranspiration (ET) in each river basin during 1982–2015 **(A–F).** The gray-shaded area represents 95% confidence interval.

Among the six basins, the largest annual ET is in the Han river basin (859 mm), and the smallest is in the Hailaer river basin (399 mm). The spatial patterns of ET in the six basins are similar to that of GPP (in section “Spatial pattern and temporal trends of vegetation growth and plant phenology”), and ET is relatively larger in the region (Supplementary Figure 4A) with a higher annual average GPP (Figure 4). There is a clear trend of increasing ET that, we found, both humid and semi-humid basins experienced the whole basin from 1982 to 2015, and the degree of increase can be divided into three levels (Figure 5 and Supplementary Figure 4B). The first level is that the increasing trend of ET in Wu river basin (mean trend = 2.1 mm/year, and 90.19% areas with increasing trends), Han river basin (3.08 mm/year, 97.54%), and Wei river basin (2.68 mm/year, 100%) are all greater than 2 mm/year; the second level is that of Fen river basin (1.86 mm/year, 89.44%), in which the increasing trend of ET is 1–2 mm/year; and the third level is that of Luan river basin (0.28 mm/year, 63.85%) and Hailaer river basin (0.47 mm/year, 82.88%), in which the increasing trend of ET is less than 1 mm/year. The increasing trend of ET in the humid region is greater than that in the semi-humid region, and there is a difference in the increasing trend of ET in the semi-humid region; the increasing trend of ET is smaller in the basins with high latitude than in the basins with low latitude.

### Spatial Pattern and Temporal Trends of Vegetation Growth and Plant Phenology

The annual GPP showed less spatial variability in the humid regions [Wu and Han river basins with mean GPP = 3.86 gC/(m^2^ day) and 4.34 gC/(m^2^ day), respectively] than that in semi-humid regions [Wei, Feng, Luan, and Hailaer river basins with mean GPP = 2.51, 2.70, 3.37, and 3.72 gC/(m^2^ day), respectively] (GPP and NDVI have similar spatial patterns, Supplementary Figure 5). And the annual GPP increased in all the river basins except Hailaer over the study period, with an average increase rate of GPP by 9.54 × 10^–3^ gC/(m^2^ day year) across all six river basins. The increase rates in GPP were reduced from humid to semi-humid river basins, and the largest increase rates of GPP were found in the Wei river basin with an average rate at 3.11 × 10 ^–2^ gC/(m^2^ day year), and the increasing GPP accounts for 93.5% of the total basin area.

The longest vegetation GSL (234 days) with the earliest SOS [day of the year (DOY) = 102] and the latest EOS (DOY = 336) was found in the Wu river basin, followed by the Han river basin. The vegetation growing season in humid river basins is longer than in semi-humid river basins. The shortest GSL was found in the Luan river basin with the latest SOS (DOY = 119) and almost earliest EOS (DOY = 305) (Table 2). Spatially, the GSL was reduced by 2.6 day per degree latitude toward north, with 0.97 days later in SOS and 1.63 days earlier in EOS (Supplementary Figure 6). Furthermore, the spring vegetation growth onset (i.e., SOS) advanced in all six river basins, and the changing rates were generally increased (i.e., less advanced rate in SOS) from southern humid river basin compared with northern semi-humid river basins, with advanced rates ranging from 0.03 days/year in Wei river basin to 0.22 days/year in Luan river basin. Opposite patterns were found in EOS, compared with SOS, where EOS delayed in all river basins except for the Luan river basin, and the longest delay was found in Wu river Basin with an average delayed rate of 0.42 days/year. Due to the advanced SOS and delayed EOS, the average GSL was extended by 4.8 to13.9 days during 1982–2015 across the six river basins (Figure 4).

**TABLE 2:** The mean phenology of the six river basins from 1982 to 2015.

River basin	SOS (DOY)	EOS (DOY)	GSL (days)
Wu	102	336	234
Han	88	329	241
Wei	102	328	226
Fen	113	303	190
Luan	119	306	186
Hailaer	113	306	192

*DOY, day of the year; SOS, start of growing season; EOS, end of growing season; GSL, growing season length.*

### Impacts of Climatic and Vegetation Factors on River Runoff

**FIGURE 6:**
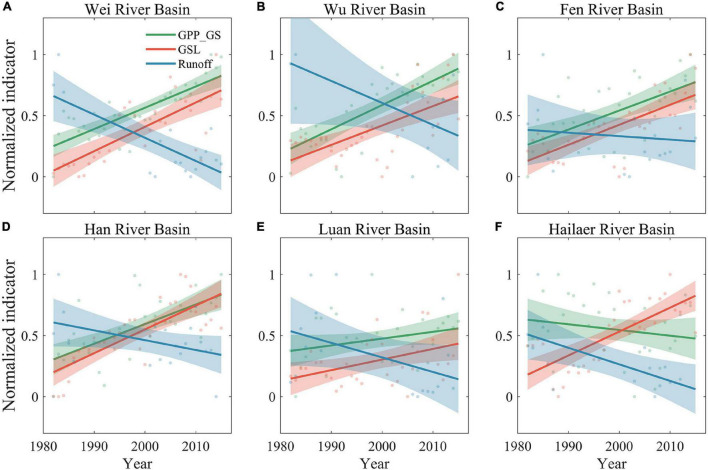
Changes of normalized runoff (Runoff, blue), growing season length (GSL, red), and the gross primary productivity during growing season (GPP_GS, green) during 1982–2015. The shadings represent 95% confidence interval. **(A)** Wei River Basin. **(B)** Wu River Basin. **(C)** Fen River Basin. **(D)** Han River Basin. **(E)** Luan River Basin. **(F)** Hailaer River Basin.

**FIGURE 7:**
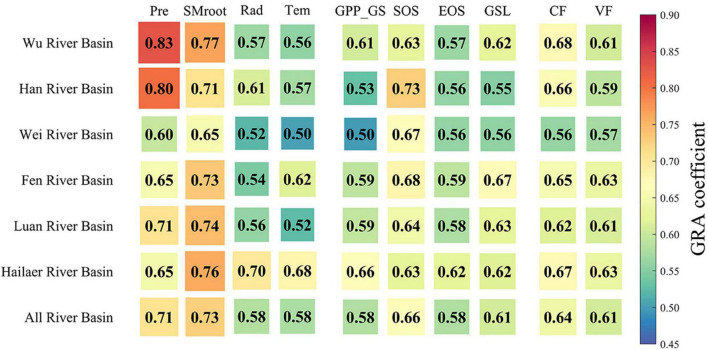
The gray relational coefficients between river runoff and the influencing factors for each river basin. Climate factors include precipitation, soil moisture in root zone (SMroot), ET, radiation, and temperature. Vegetation factors include GPP during growing season, start of growing season, end of growing season, and GSL.

Across all six river basins, the river runoff showed different degrees of decrease (two river basins showed a significant trend, three basins showed a marginally significant trend, and one basin didn’t show a significant trend), while GPP_GS (except the Hailaer river basin) and GSL showed an increase and delay trend during 1982–2015, respectively (Figures 3, 6). The GRA results showed that the coefficients of climate and vegetation on runoffs are similar, which are 0.64 and 0.61 for CF and VF, respectively, across all six river basins (Figure 7). For each factor, we found that the precipitation (Pre) and soil moisture (SMroot) are the most important CFs affecting river runoff, with an average gray relational coefficient of 0.71 and 0.73 for Pre and SMroot, respectively. Interestingly, the effect of VFs, i.e., GPP_GS (*r* = 0.59), SOS (*r* = 0.65), EOS (*r* = 0.59), and GSL (*r* = 0.61), on runoffs is larger than temperature (*r* = 0.56) and radiation (*r* = 0.59) (Figure 7).

**FIGURE 8:**
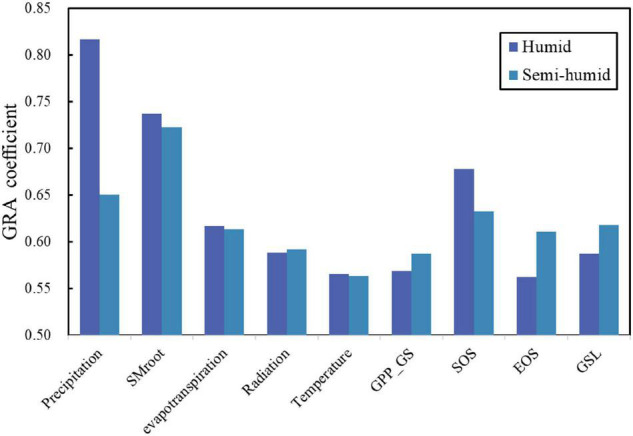
The gray relational coefficients between river runoff and the influencing factors for humid (deep blue) and semi-humid regions (light blue**).** SMroot, GPP, SOS, EOS, and GSL.

We further estimated the mean GRA coefficients of each climate and VF for humid and semi-humid river basins (Figures 7, 8). Consistently, we found the largest two factors, i.e., Pre and SMroot, for both humid and semi-humid river basins. Especially, the Pre determined the runoffs in the humid river basins with average GRA coefficients *r* = 0.82, and the SMroot is the dominant factor in semi-humid river basins (*r* = 0.72). The third important factor is the SOS for both humid and semi-humid river basins with average *r* = 0.68 and 0.63, respectively. For the VFs, we found that the spring phenology SOS (*r* = 0.65) largely impacted the runoff in humid river basins, but the autumn phenology EOS (*r* = 0.61) mainly impacted the runoff in semi-humid river basins. Overall, we found that the phenological factors impacted the runoffs as the secondary dominators, and the spring phenology mainly impacted the humid regions, but both the spring and autumn phenology mainly impacted the semi-humid regions.

## Discussion

### Temporal–Spatial Variation of Vegetation Growth and Phenology

Climate warming promotes vegetation growth, as long as water and nutrients are not limited (Wu et al., 2003; Xu et al., 2018). The larger increase in GPP in the humid regions than in semi-humid regions in this study could be partially linked to water availability for plant growth. The reduced trend of GPP found in the Hailaer river basin could be caused by land-use change, e.g., deforestation and cropland expansion over the study period, according to the previous reports (Ye et al., 2009; Deng et al., 2010). For the vegetation phenology, we found large spatial heterogeneity but consistent temporal patterns, i.e., earlier SOS and later EOS resulted in longer GSL, which is in line with previous studies, and a warming spring could help vegetations meet their forcing demands earlier and more accumulation of GPP would prolong the life of leaves and thus delay the EOS (Piao et al., 2019; Geng et al., 2020). Interestingly, we further found that the advancing rates of SOS were larger in northern semi-humid regions than southern humid regions where a larger delayed rate of EOS was found over the study period of 1982–2015. That might be because the vegetation in high-latitude regions is more sensitive to spring temperature variation, which is an adaptive protection mechanism in plants (Prevéy et al., 2017), but the EOS might be more sensitive to light conditions and/or phycological growth cues (Zani et al., 2020, 2021). Overall, we found the extended GSL for river basins in the semi-humid region was mainly attributed to the earlier SOS, but the delayed EOS contributed mostly to the prolonged GSL for river basins in humid regions.

### Variation of River Runoff and Its Difference by Regions

We found decreasing trend of runoff across all six river basins in China, which is in line with previous studies (Goulden and Bales, 2014; Lei et al., 2014; Xu et al., 2019; Zhang et al., 2019). The reasons may be related to the fact that the global warming-induced increases in vegetation growth and prolonged growing season enhanced the precipitation interception, plant water use, vegetation transpiration during the growing season (McNaughton and Jarvis, 1983; Goulden and Bales, 2014; Hwang et al., 2018; Kim et al., 2018; Geng et al., 2020), and increased water demand for vegetation growth, which could cause the decreased river runoff (Hwang et al., 2018; Kim et al., 2018). While other studies suggest that, with global warming, the water cycle on land has been accelerated, the water vapor transmission pattern in the atmosphere has been changed, and the runoff in different areas has different responses to vegetation change (Lian et al., 2020). In addition, the runoff reduction was greater in semi-humid regions than that in the humid region, which might be because a larger portion of precipitation was used for ET in the semi-humid region. We found that, in the humid region, river runoff was mainly controlled by CFs, especially precipitation; however, the VFs played more important roles in the semi-humid region, and similar results were also previously documented (Liu M. et al., 2018; Zhang et al., 2019).

Although precipitation was the most important factor affecting river runoff in both humid and semi-humid regions, we found that the impacts of vegetation phenology on runoff can also be seen, and the impacts were different between humid and semi-humid regions. In detail, spring phenology mainly affected the runoff in humid river basins, but both spring and autumn phenology affected the runoff in semi-humid river basins. The larger values of GPP_GS are associated with larger water loss (ET) (Suzuki et al., 2007). In the humid region, the advanced SOS increased vegetation water demand during the early spring (Hufkens et al., 2016; Jin et al., 2017), which plays a more important role in causing runoff reduction in the humid region. Compared with the humid regions, the SOS was also advanced in semi-humid regions, and the time in spring is generally a dry season in northern China; therefore, the early start of growth likely results in a water shortage for plant growth and reduction of soil moisture in summer and induces a legacy effect on river runoff (Piao et al., 2019; Zhou et al., 2020). In autumn, the temperature is suitable for vegetation growth, and the delayed phenology likely prolongs the life of plant leaves and finally results in large ET in the semi-humid river basins. Therefore, spring and autumn phenology likely mainly affected the runoff in the semi-humid river basins, and the spring phenology likely mainly affected the runoff in the humid river basins.

### Limitation

The observed runoff data were obtained from the hydrological stations; although we selected river basins to try to avoid human activities effects (land-use change), differences between observed runoff and natural runoff (the effect of land-use change) are difficult to rule out; however, during 1980–2015, the change of land use in the study basins was small, and the proportion of various land uses remained basically unchanged (Supplementary Table 2). We assumed that could not be the main reason for the river runoffs changes, and the change of runoff in the study period was mainly caused by the influence of climate and environmental factors, but impacts of human activities (land-use change) cannot be ignored (Ye et al., 2003; Du and Shi, 2012; Wang et al., 2013; Chang et al., 2015). Meanwhile, the recording gap of time series may bring greater uncertainty to the quantification of runoff trends in the basin. And we hoped that our future research can conduct more accurate research estimated using the model approaches to re-estimate the runoffs data during the missing periods. However, it is still a challenge to study the model that can accurately simulate the annual and inter-annual growth fluctuations (phenology changes and greening) of vegetation. So, the processes of vegetation phenology, and the underlying mechanisms affecting river runoffs, need further investigations (Guo et al., 2021) and to be accurately formulated and coupled with hydrological models to better simulate vegetation dynamic, e.g., LAI, and understand the response of hydrological processes to ongoing climate change in future.

## Conclusion

In this study, six river basins in the humid and semi-humid regions in China were selected to analyze the temporal changes in runoff and vegetation dynamics during 1982–2015. We found that the observed runoff decreased in all basins, and the decrease in semi-humid area was larger than that in the humid area. The vegetation in all basins showed a greening trend, with a larger increase in the humid area than that in the semi-humid area, and the length of the growing season has also increased (1.4–4.1 days per decade). In the humid region, the extension of the growing season was mainly due to the delayed autumn phenology, while in the semi-humid region, the extension of the growing season was mainly due to the advanced spring phenology. The GRA results suggested that precipitation and soil moisture content are the dominant factors affecting runoff in both humid and semi-humid river basins, but VFs (GPP in growing season and phenology) are the secondly important factors, similar to or larger than ET, which are highly associated with plant factors, larger than temperature. Meanwhile, the impacts of phenology factors on runoff were different across different hydroclimatic zones. The spring phenology is the main VF in the humid region, while spring and autumn phenology are the main VFs in the semi-humid region. Overall, this study revealed the potential influences of climatic and VFs on river runoff in different hydroclimatic regions, which provide insights into hydrological processes between humid and semi-humid regions.

## Data Availability Statement

The original contributions presented in the study are included in the article/Supplementary Material, further inquiries can be directed to the corresponding authors.

## Author Contributions

YF: conceptualization, writing—reviewing and editing, project administration, and funding acquisition. FH: conceptualization. SC: methodology, software, formal analysis, resources, and writing—original draft preparation. XZ, ZX, and ZH: validation. XG: investigation. JT: data curation, visualization, and supervision. All authors have read and agreed to the published version of this manuscript.

## Conflict of Interest

The authors declare that the research was conducted in the absence of any commercial or financial relationships that could be construed as a potential conflict of interest.

## Publisher’s Note

All claims expressed in this article are solely those of the authors and do not necessarily represent those of their affiliated organizations, or those of the publisher, the editors and the reviewers. Any product that may be evaluated in this article, or claim that may be made by its manufacturer, is not guaranteed or endorsed by the publisher.

## Footnotes

1
http://data.tpdc.ac.cn/

2
https://www.nasa.gov/nex

3
http://glass-product.bnu.edu.cn/

4
https://vip.arizona.edu

5
www.GLEAM.eu

6
www.resdc.cn
