# Supplementary material for: Influences of Shifted Vegetation Phenology on Runoff Across a Hydroclimatic Gradient
Source: Front Plant Sci. 2022 Jan 4;12:802664. doi: 10.3389/fpls.2021.802664 (PMC8764410; doi:10.3389/fpls.2021.802664)
Supplement: Supplementary file 1 [file Data_Sheet_1.docx]

Supplementary Material

# Supplementary Figures and Tables

## Supplementary Figures


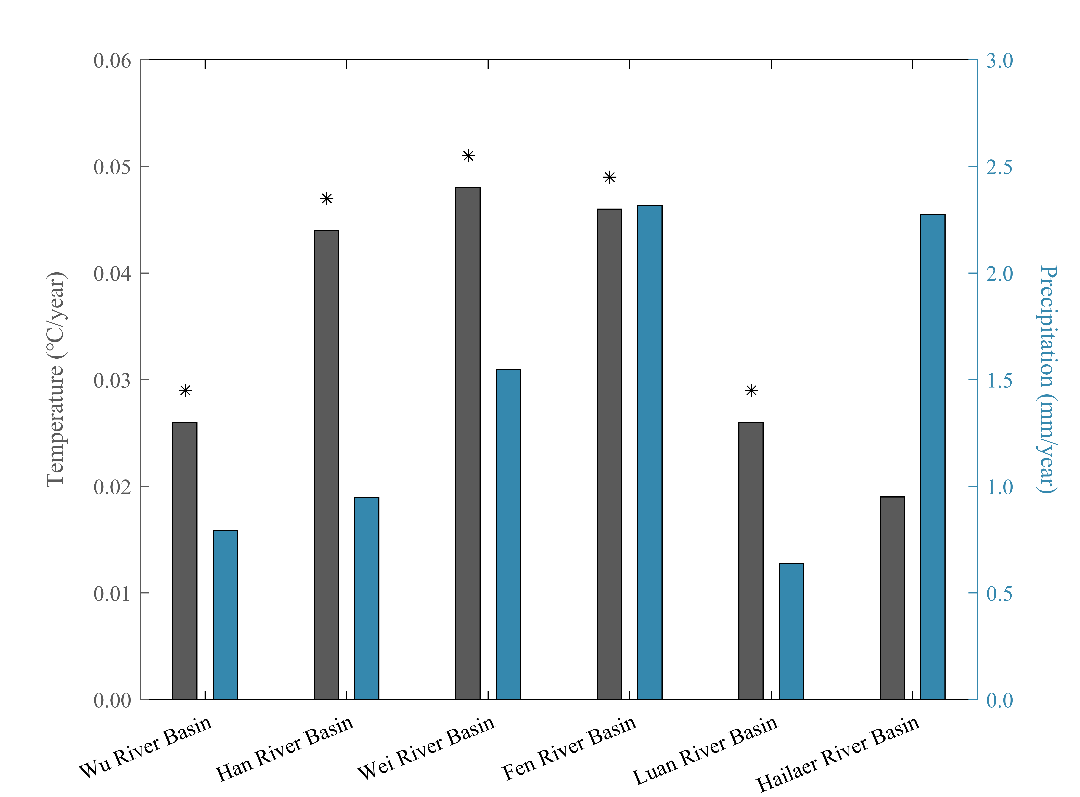


**Figure S1. The slopes of the linear regression of climatic factors of six river basins.** The black stars above bars indicate significant level at P<0.05.

**
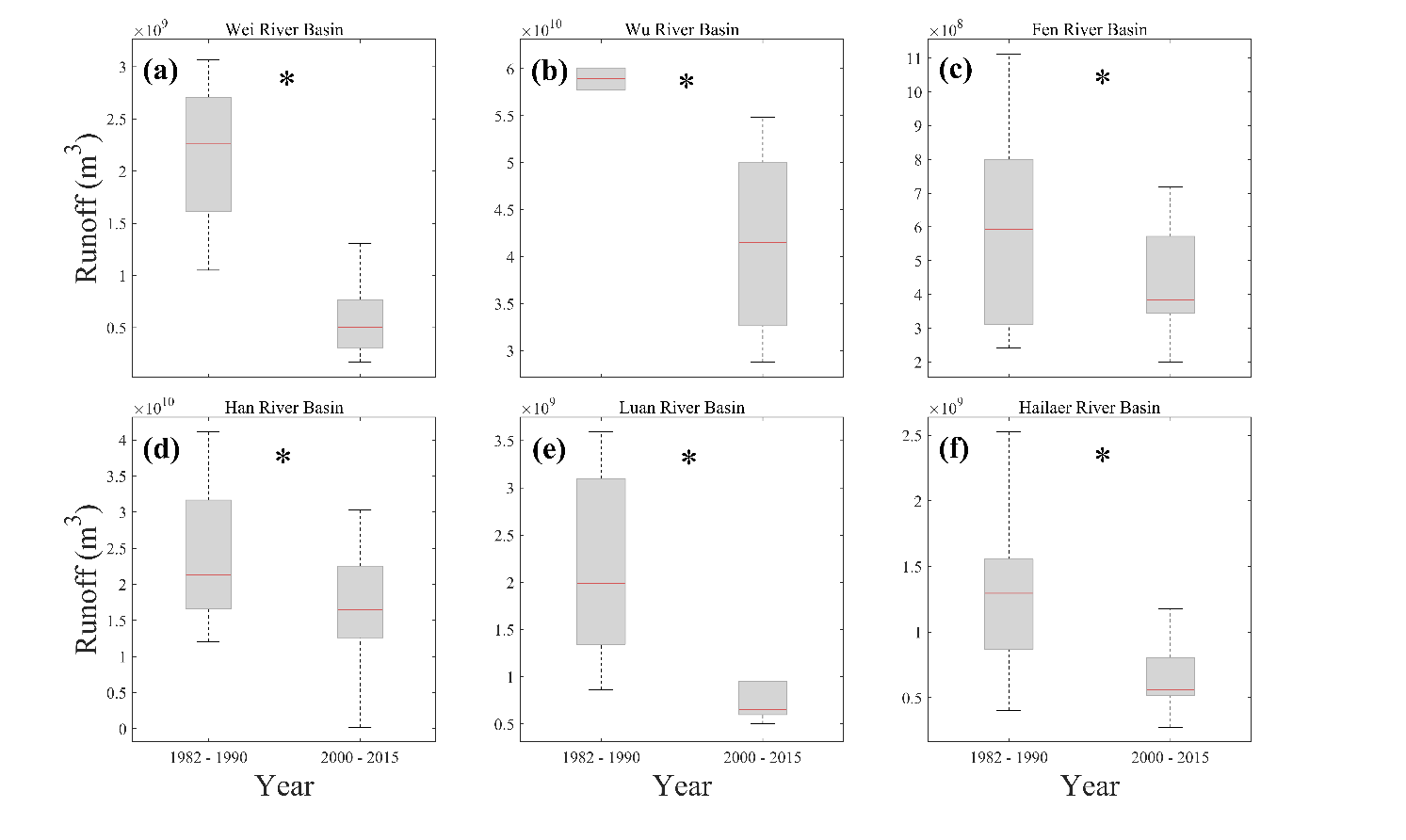
**

**Figure S2. the mean runoffs between the periods before 1990 and after 2000.**


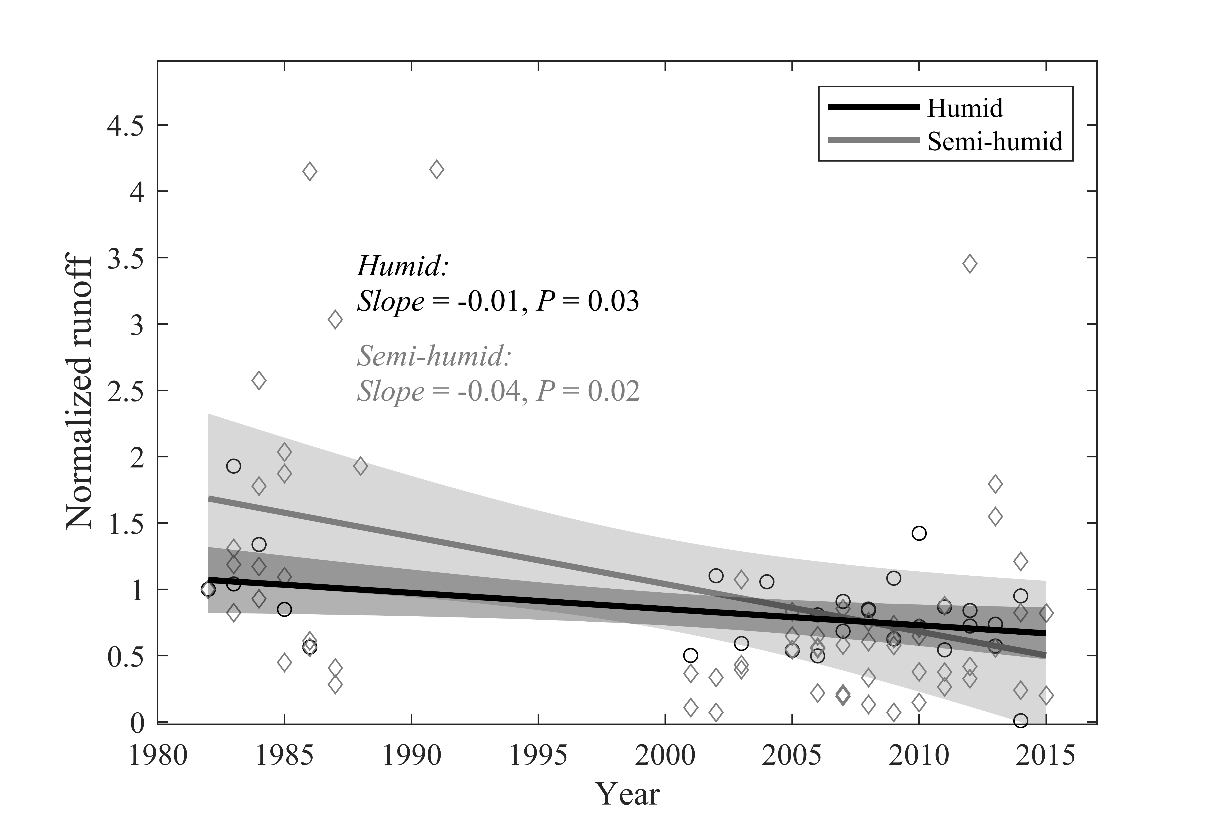


**Figure S3. Temporal changes of normalized runoff in humid (Han and Wu river basins, deep black) and semi-humid (Wei, Fen, Luan and Hailaer river basins, grey) basins during 1982-2015.** The grey-shaded area represents 95% confidence interval.


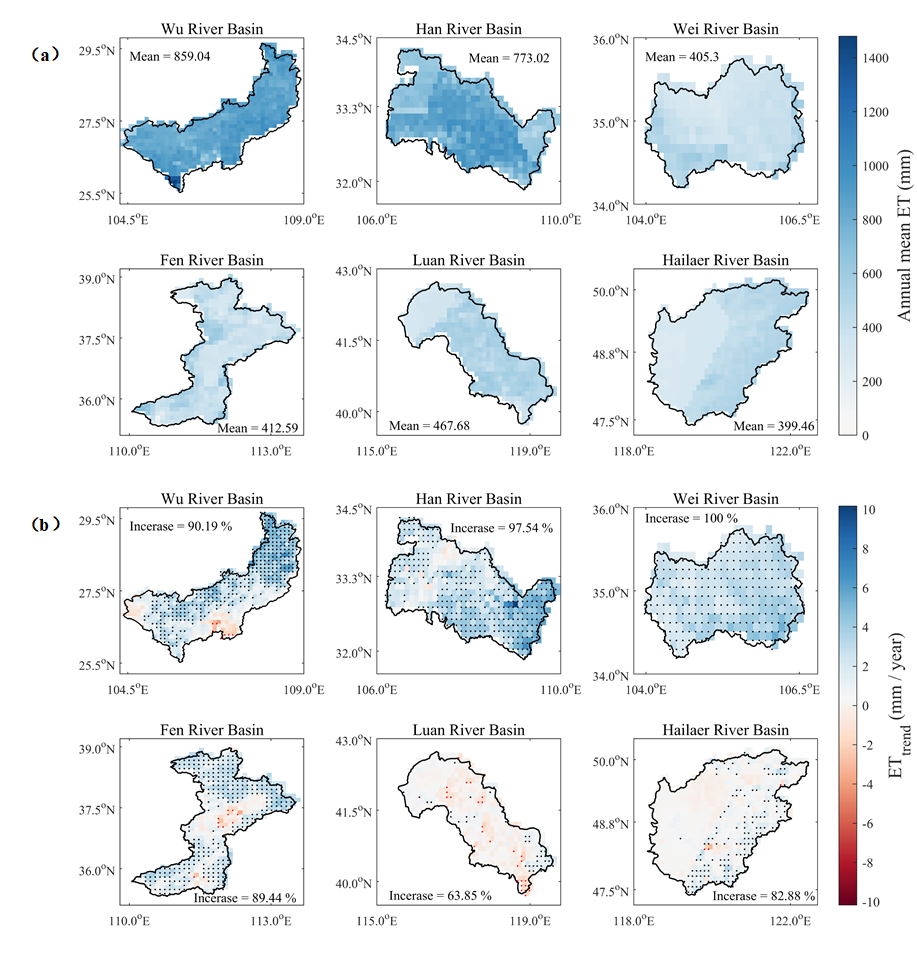


**Figure S4. Spatial patterns of multi-year average ET (evapotranspiration) and change trend over the period 1982-2015 of the six river basins.** Black scatters denote indicate a significant increase in ET, and red scatters for a significant decrease.


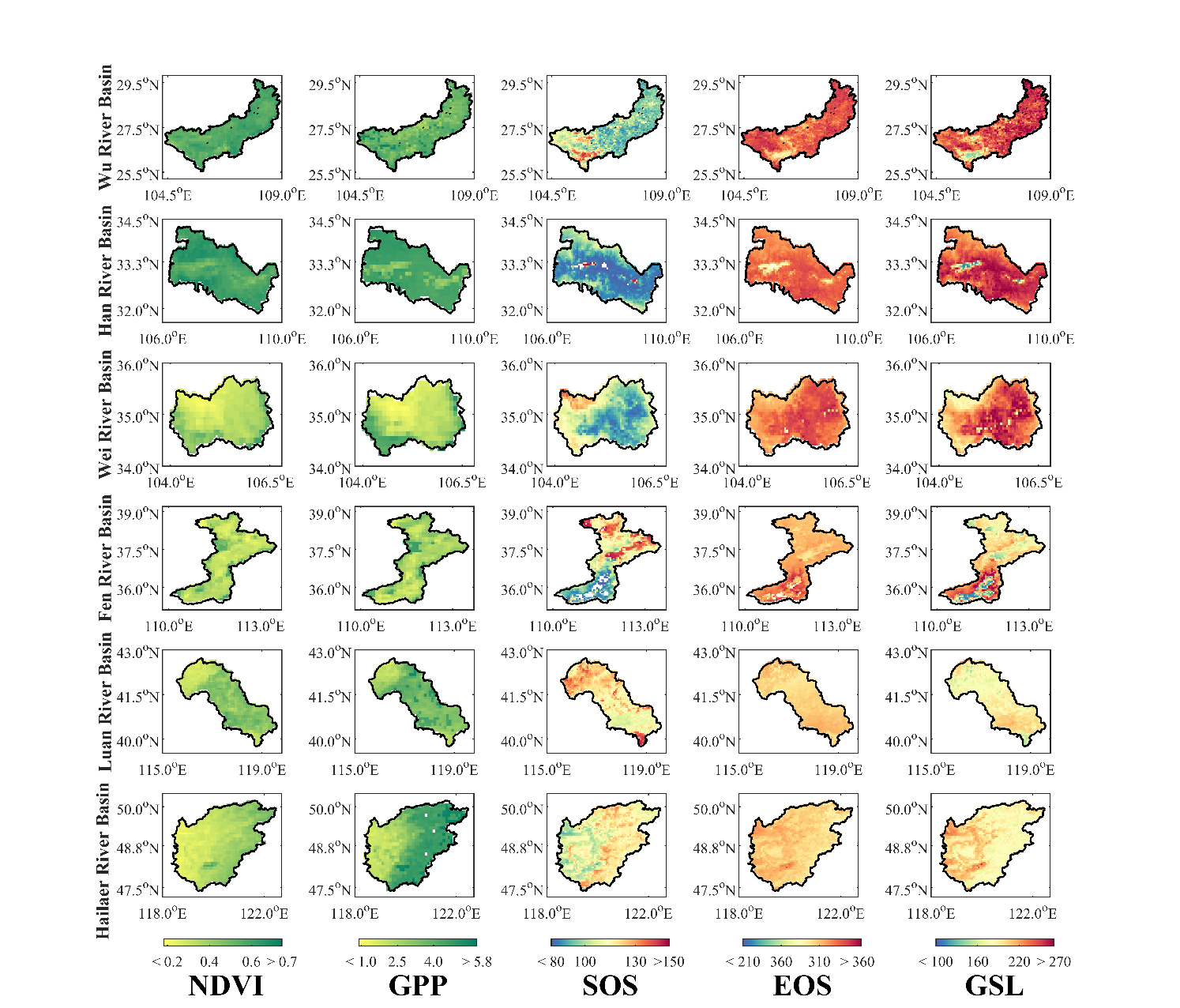


**Figure S5. Spatial patterns of mean vegetation growth (here, used normalized differential vegetation index, NDVI , Gross Primary Productivity, GPP, gC / (m2 day),) and phenological indices over the period 1982-2015 of the six river basins.** SOS, start of growing season; EOS, end of growing season; GSL, growing season length.

**
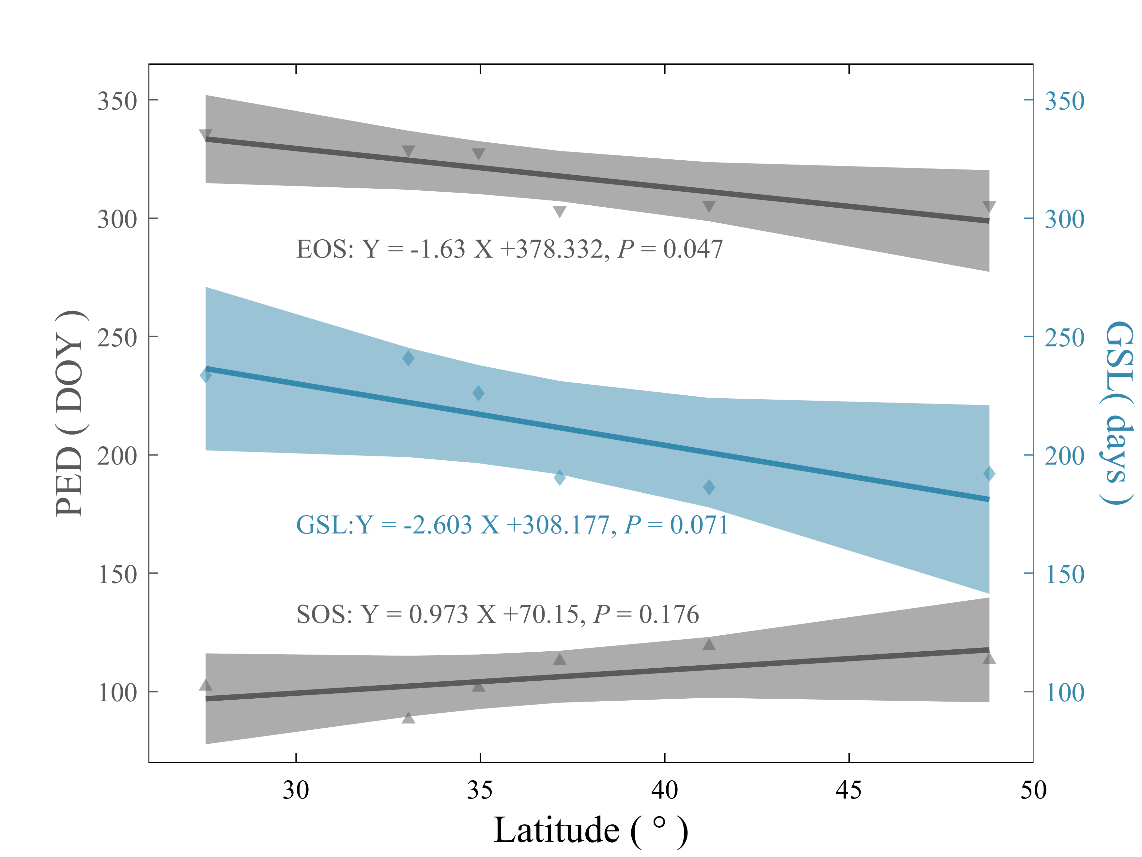
**

**Figure S6. Latitudinal changes of phenological dates across the six basins.** PED, phenological events date, SOS, start of growing season; EOS, end of growing season; GSL, growing season length. The upper triangle represents SOS, the lower triangle represents EOS and the diamond represents GSL. The grey-shaded area represents 95% confidence interval.

## Supplementary Tables

**Table S1 The results of analysis of covariance.** Dependent Variable: Runoff depth (mm), region, humid region and semi-humid region.

| Source | Type III Sum of Squares | df | Mean Square | F | Sig. | Partial Eta Squared |
| --- | --- | --- | --- | --- | --- | --- |
| Corrected Model | 4590660.453 | 3 | 1530220.151 | 115.406 | .000 | .788 |
| Intercept | 114474.121 | 1 | 114474.121 | 8.633 | .004 | .085 |
| year | 105706.238 | 1 | 105706.238 | 7.972 | .006 | .079 |
| region | 62374.164 | 1 | 62374.164 | 4.704 | .033 | .048 |
| region * year | 56522.886 | 1 | 56522.886 | 4.263 | .042 | .044 |
| Error | 1233124.162 | 93 | 13259.400 |  |  |  |
| Total | 8590179.341 | 97 |  |  |  |  |
| Corrected Total | 5823784.615 | 96 |  |  |  |  |

**a. R Squared = .788 (Adjusted R Squared = .781)**

**Table S2 Land use from 1980 to 2015 of six river basins.** Others include Sand, Gobi, Saline and alkaline land, wetland and Bare land.

| basin | Land use type (Km^2^) | | | | | | |
| --- | --- | --- | --- | --- | --- | --- | --- |
|  | year | cropland | forest | grass | water | urban | others |
| Wu | 1980 | 21433 | 34847 | 12713 | 262 | 306 | 8 |
|  | 1990 | 21388 | 34827 | 12722 | 274 | 329 | 12 |
|  | 1995 | 21393 | 34811 | 12730 | 275 | 331 | 12 |
|  | 2000 | 21636 | 34252 | 13024 | 276 | 352 | 12 |
|  | 2005 | 21736 | 34602 | 12558 | 279 | 366 | 11 |
|  | 2015 | 21315 | 34478 | 12483 | 309 | 958 | 9 |
| Han | 1980 | 11945 | 15272 | 19114 | 216 | 237 | 4 |
|  | 1990 | 11973 | 15231 | 19084 | 202 | 239 | 4 |
|  | 1995 | 11940 | 15220 | 19134 | 200 | 235 | 4 |
|  | 2000 | 11992 | 15218 | 19071 | 201 | 247 | 4 |
|  | 2005 | 11904 | 15229 | 19105 | 208 | 283 | 4 |
|  | 2015 | 11842 | 15227 | 19105 | 218 | 332 | 9 |
| Wei | 1980 | 12254 | 2643 | 10506 | 133 | 507 | 86 |
|  | 1990 | 12253 | 2644 | 10453 | 128 | 517 | 85 |
|  | 1995 | 12287 | 2630 | 10426 | 126 | 525 | 86 |
|  | 2000 | 12285 | 2649 | 10397 | 127 | 537 | 85 |
|  | 2005 | 12090 | 2662 | 10553 | 128 | 562 | 85 |
|  | 2015 | 11933 | 2659 | 10575 | 137 | 664 | 113 |
| Fen | 1980 | 18371 | 13486 | 12102 | 509 | 1489 | 7 |
|  | 1990 | 18350 | 13465 | 12047 | 520 | 1572 | 10 |
|  | 1995 | 18325 | 13545 | 11855 | 566 | 1662 | 11 |
|  | 2000 | 18331 | 13457 | 11970 | 497 | 1698 | 11 |
|  | 2005 | 18212 | 13452 | 11989 | 504 | 1796 | 11 |
|  | 2015 | 18028 | 13451 | 11961 | 490 | 2024 | 10 |
| Luan | 1980 | 9861 | 17132 | 14019 | 635 | 479 | 1385 |
|  | 1990 | 9850 | 17044 | 14036 | 626 | 501 | 1380 |
|  | 1995 | 9999 | 16924 | 13993 | 631 | 511 | 1379 |
|  | 2000 | 10041 | 16961 | 13861 | 633 | 518 | 1423 |
|  | 2005 | 10016 | 16952 | 13811 | 648 | 562 | 1448 |
|  | 2015 | 9949 | 16943 | 13950 | 654 | 630 | 1311 |
| Hailaer | 1980 | 4042 | 15527 | 31230 | 359 | 148 | 3220 |
|  | 1990 | 4222 | 15484 | 31088 | 380 | 208 | 3142 |
|  | 1995 | 3295 | 15612 | 31909 | 365 | 211 | 3132 |
|  | 2000 | 3488 | 15455 | 31815 | 381 | 211 | 3174 |
|  | 2005 | 3560 | 15549 | 31735 | 373 | 211 | 3096 |
|  | 2015 | 3585 | 15545 | 31662 | 377 | 261 | 3094 |

**Table S3 Acronyms in this paper and their full names.**

| Acronyms | Full name |
| --- | --- |
| SOS | Start of growing season |
| EOS | End of growing season |
| GSL | Growing season length |
| NDVI | Normalized Difference Vegetation Index |
| GPP | Gross primary productivity |
| MAT | Mean annual temperature |
| MAP | mean annual precipitation |
